# Sunlight‐Induced Lipid Oxidation and Degradation of Edible Oil Mediated by Hemoglobin: Implications for Food System Sustainability

**DOI:** 10.1002/fsn3.72423

**Published:** 2026-09-28

**Authors:** Mahdi Hajimohammadi, Fadhel H. Faraj, Mona Boghdachi, Asawer A. Alwasiti, Zainab Y. Shnain

**Affiliations:** ^1^ Faculty of Chemistry Kharazmi University Tehran Iran; ^2^ Chemical Engineering College University of Technology Baghdad Iraq

**Keywords:** edible oil, food sustainability, heme‐mediated oxidation, hemoglobin, lipid oxidation, singlet oxygen

## Abstract

Lipid oxidation is one of the major factors affecting the quality, safety, and shelf life of edible oils. In this study, sunlight‐induced oxidation of edible oil was investigated in an aqueous system using erythrocyte‐derived hemoglobin (Hb) as a natural biocatalyst. Significant oxidation was observed only under visible‐light irradiation in the presence of Hb and oxygen. The peroxide value (PV) increased rapidly, reaching 9.7 meq O_2_/kg within 2.5 h, followed by the degradation of unsaturated fatty acids, indicating the occurrence of secondary oxidation reactions. Proton nuclear magnetic resonance (^1^H NMR) analysis showed a 31.2% decrease in vinylic protons and a 36.9% decrease in bis‐allylic protons, together with the appearance of an aldehydic signal at *δ* 9.5 ppm, confirming the formation of malondialdehyde (MDA). The generation of singlet oxygen (^1^O_2_) was supported by anthracene bleaching (33%) and its inhibition by sodium azide (NaN_3_), while the inhibitory effect of butylated hydroxytoluene (BHT) suggested the simultaneous involvement of radical‐mediated oxidation pathways. Compared with Rose Bengal and potassium permanganate (KMnO_4_), Hb showed higher photodegradation activity, likely due to the combined contribution of singlet oxygen generation and ferryl‐mediated (HbFe^(IV)^═O) hydrogen abstraction. Beyond advancing our understanding of the still underexplored mechanism of hemoglobin‐mediated photooxidation, these findings provide a scientific basis for exploring food‐derived hemoproteins as naturally abundant photosensitizers for the treatment of lipid‐rich wastewater.

## Graphical abstract

Sunlight‐activated hemoglobin (Hb) functions as a robust natural biocatalyst that promotes the degradation of edible oils through a dual, synergistic pathway. Photo‐excitation of Hb triggers simultaneous energy transfer, generating singlet oxygen (^1^O_2_) and electron transfer, forming highly reactive ferryl (HbFe^(IV)^═O) species. This mechanistic interplay accelerates lipid peroxidation and chain scission, leading to significant malondialdehyde (MDA) accumulation. These findings elucidate the underexplored hemoprotein mediated photooxidation mechanism, providing critical insights into food oil stability and potential strategies for the treatment of lipid‐rich wastewater.

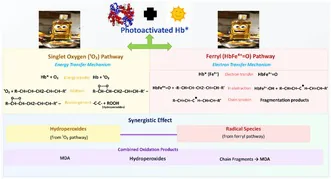

## 1 Introduction

Lipid oxidation and photooxidation are recognized as major factors influencing the quality, nutritional value, and shelf life of edible oils and other lipid‐containing foods (Looper and Vierck 2023; MacDougall 1982; Machado et al. 2023; Sebranek and Bacus 2007; Talbot 2016; Versino et al. 2023). Oxidative deterioration of lipids results in the formation of hydroperoxides, aldehydes, and other secondary oxidation products that adversely affect flavor, safety, and consumer acceptability (Agnez‐Lima et al. 2012; Domínguez et al. 2019; Droge 2002; Xi Huang and Ahn 2019; Kasaai 2025; Shetty et al. 2026). Among these oxidation markers, PV and MDA formation are widely recognized as key indicators of the primary and advanced stages of lipid peroxidation, respectively, in food systems (Abeyrathne et al. 2021). Since lipid photooxidation represents one of the major pathways contributing to oxidative deterioration, understanding its underlying mechanisms is essential for developing effective strategies to enhance food stability, optimize storage conditions, and improve processing practices (Afonso et al. 1999; Min and Boff 2002; Papuc et al. 2017).

In addition to storage‐related oxidation, edible oil processing and food manufacturing generate lipid‐rich effluents that pose significant environmental and technological challenges (Ahmad et al. 2020; Dordević et al. 2025; Jiang et al. 2015; Narra and Shahpasand 2025; Urrutia et al. 2024; C. Wang et al. 2025; Zhao et al. 2024). Conventional oxidation‐based treatments, such as ozonation and Fenton processes, can effectively degrade organic pollutants; however, they often require harsh chemical oxidants and energy‐intensive operating conditions (Alatabe et al. 2024; Ayoub 2022; Gogate and Pandit 2004; Malato et al. 2009). Consequently, there is growing interest in sustainable light‐driven strategies that harness naturally occurring biomolecules for environmentally friendly applications.

Hemoglobin, a heme‐containing protein abundant in animal tissues and slaughterhouse by‐products, is traditionally recognized for its role in oxygen transport in muscle foods (Nagababu and Rifkind 2004). However, heme proteins are also recognized as important mediators of lipid oxidation in meat and other food matrices (Meunier 2000). The heme prosthetic group, composed of an iron‐centered porphyrin structure, can participate in redox reactions that facilitate the generation and propagation of lipid radicals (Minning et al. 1999). Under light exposure, heme systems may further generate reactive oxygen species, including singlet oxygen, thereby accelerating oxidative processes (De Jager et al. 2017; Hajimohammadi, Verjani, et al. 2018). Previous studies have demonstrated that porphyrin and hemoglobin‐based systems can promote aerobic photooxidation of fatty acids and lipids under visible light (Hajimohammadi and Moradi 2025; Hajimohammadi and Nosrati 2018; Hajimohammadi et al. 2024; Hajimohammadi, Vaziri Sereshk, et al. 2018; Wu et al. 2024).

Building on these considerations, the present work aimed to elucidate the mechanism of sunlight‐induced lipid oxidation mediated by erythrocyte‐derived hemoglobin in an aqueous edible oil system. Its novelty lies in using structurally intact, erythrocyte‐derived hemoglobin as a native photosensitizer at the aqueous–oil interface under sunlight and bridging mechanistic photochemistry with biomimetic conditions. The work integrates singlet oxygen and radical/heme‐driven pathways within a single native system under visible light, enabling evaluation of their combined roles in lipid degradation. In addition to improving our understanding of the still underexplored mechanism of hemoglobin‐mediated photooxidation, this study provides a basis for exploring blood‐derived proteins as low‐cost, naturally abundant photosensitizers for the future treatment of lipid‐rich wastewater from edible oil processing.

Based on scavenger assays and spectroscopic analyses, we proposed that hemoglobin promotes lipid oxidation through a dual oxidative mechanism involving (i) singlet oxygen generation via energy transfer and (ii) a tentative heme iron‐mediated radical pathway. The combined contribution of these oxidative processes results in substantial degradation of unsaturated fatty acids under mild conditions. By connecting fundamental lipid oxidation chemistry with practical considerations in food stability and sustainable processing, this study advances the understanding of heme‐mediated oxidative reactions beyond their traditional biological functions.

## 2 Materials and Methods

### 2.1 Materials

Rose Bengal, anthracene, BHT, KMnO_4_, NaN_3_, acetonitrile (CH_3_CN), ethanol (EtOH), acetone ((CH_3_)_2_CO), methanol (MeOH), and dimethyl sulfoxide (DMSO) were purchased from Fluka and Merck and used as received without further purification. Commercially available edible oil (Oila, canola oil) was purchased from a local market and used without additional purification.

### 2.2 Preparation of Erythrocytes

Erythrocyte membranes were prepared according to the standard method described by Dodge et al. (1963). Defibrinated sheep blood was obtained from Kimiakavoshazma Company (Iran). Upon arrival, the samples were stored at 4°C and used within 48 h. Whole blood was centrifuged at 3000 rpm for 10 min at 4°C to separate the erythrocyte fraction from plasma. The collected erythrocytes were washed three times with phosphate‐buffered saline (PBS; 10 mM, pH 7.4) and subsequently resuspended in phosphate buffer (10 mM, pH 7.4). The erythrocyte suspension was adjusted to a protein concentration of approximately 1.0 mg mL^−1^ and freshly prepared suspensions were used for all photodegradation experiments. Hemoglobin concentration was normalized before each experiment by adjusting all samples to the same protein concentration using phosphate buffer (10 mM, pH 7.4). The hemoglobin concentration was further verified spectrophotometrically based on the Soret absorption band prior to analysis using a Shimadzu UV–visible spectrophotometer (UV‐2100) at 407 nm.

### 2.3 Photodegradation Procedure

A solution containing edible oil (2 × 10^−2^ v/v) and erythrocyte suspension (2 × 10^−2^ v/v) was irradiated using a solar simulator equipped with 276 LED lamps (1 W, 2.3 V; 380–780 nm; 59,660 lx; 8.74 mW cm^−2^) for 2.5 h at 25°C under atmospheric pressure in a water/acetonitrile (9:1, v/v) system. During irradiation, air was continuously bubbled into the reaction mixture through a glass diffuser to maintain aerobic conditions. Air was supplied at ambient pressure (1 atm) at an approximate flow rate of 50 mL min^−1^, while the reaction mixture was magnetically stirred throughout the experiment. Control experiments were conducted in the dark and in the absence of Hb. Aliquots were collected at defined time intervals for subsequent UV–Vis analysis. PVs were determined using the iodometric method described by Barthel and Grosch (1974) and expressed as meq O_2_ kg^−1^ oil. Instrument performance and analytical consistency were verified using standard peroxide solutions prior to analysis. Erythrocyte degradation was monitored using a Shimadzu UV–visible spectrophotometer (UV‐2100) at 407 nm. UV–visible absorption spectra were recorded with the same instrument equipped with 1 cm quartz cuvettes over the wavelength range of 200–800 nm at a spectral resolution of 1 nm. Baseline correction was performed using the corresponding solvent mixture as the reference.

### 2.4 Preparation of Samples for ^1^H NMR Analysis

A solution of edible oil (2 × 10^−2^ v/v) and erythrocyte suspension (2 × 10^−2^ v/v) was irradiated under the same conditions described in Section 2.3 for 6 h. ^1^H NMR spectra were recorded on a Bruker AMX 300 MHz spectrometer at 25°C using tetramethylsilane (TMS) as the internal standard. Spectra were acquired using a standard single‐pulse sequence with 32 scans, a relaxation delay of 1 s, and appropriate spectral width for proton detection. Chemical shifts are reported in ppm relative to TMS. The appearance of aldehydic resonances in the region of 9.4–9.6 ppm was used to confirm the formation of MDA.

### 2.5 Sample Preparation for Anthracene Bleaching Test

A solution containing anthracene (4 × 10^−4^ M), edible oil (2 × 10^−2^ v/v), and erythrocyte suspension (2 × 10^−2^ v/v) was irradiated under the same conditions described in Section 2.3, for 4 h. The resulting products were analyzed using a Shimadzu UV–visible spectrophotometer (UV‐2100) at 375 nm.

### 2.6 Sample Preparation for Investigation of Solvent Effects on Photooxidation Efficiency

A solution containing edible oil (2 × 10^−2^ v/v) and erythrocyte suspension (2 × 10^−2^ v/v) was irradiated under the same conditions described in Section 2.3, for 2.5 h in a water/solvent (9:1, v/v) system.

### 2.7 Effect of Standard Oxidants on Photooxidation Efficiency

Edible oil (2 × 10^−2^ v/v) was separately combined with erythrocyte suspension (2 × 10^−2^ v/v) or Rose Bengal (1 × 10^−4^ M) as a reference photosensitizer and then irradiated under the same conditions described in Section 2.3 for 2.5 h. In parallel, the reaction containing edible oil (2 × 10^−2^ v/v) and KMnO_4_ (1 × 10^−4^ M) was run under similar conditions but kept in the dark.

### 2.8 Statistical Analysis

All experiments were performed independently in triplicate (*n* = 3). Results were expressed as mean ± standard deviation (SD). Statistical analyses were conducted using SAS software (Version 9.4, SAS Institute Inc., Cary, NC, USA). Differences among experimental groups were assessed by one‐way analysis of variance (ANOVA), followed by Duncan's multiple range test for post hoc comparisons. Statistical significance was set at *p* < 0.05. In figures, different uppercase letters indicate statistically significant differences among groups based on Duncan's test.

## 3 Results

### 3.1 Photocatalytic Oxidation of Edible Oil Under Sunlight

Erythrocytes were employed in this study as a natural hemoglobin‐containing system that provides an intrinsic and biocompatible photosensitizing environment. The use of intact or lysed erythrocytes offers a physiologically relevant matrix for hemoglobin, allowing the preservation of its native structure and redox properties during light irradiation (Hajimohammadi et al. 2024; Wu et al. 2024). This strategy enables the direct investigation of hemoglobin‐mediated photodegradation of edible oils in aqueous systems under conditions that more closely resemble biological and environmental environments. Thus, erythrocytes serve not only as a natural source of hemoglobin but also as a functional model. The edible oil/Hb system was examined to elucidate the dual‐function photooxidative mechanisms driven by sunlight. Following 24 h of simulated sunlight irradiation, the edible oil/Hb mixture showed a gradual decrease in turbidity and yellow coloration. UV–Vis analysis revealed a reduction in the characteristic Soret absorption band of the heme‐containing system, corresponding to a 69.8% decrease in Soret‐band intensity and indicating substantial photochemical changes in the Hb chromophore during irradiation (Figure 1a). Control experiments performed (i) in the dark, (ii) in the absence of Hb, and (iii) under oxygen‐limited conditions showed negligible oxidation, demonstrating that the simultaneous presence of hemoglobin, oxygen, and light is required for efficient photooxidation (Table 1, Entries 1–4). These results confirm the essential contribution of Hb as a photosensitizer in the light‐driven degradation of edible oil. Furthermore, the similarity between the emission spectrum of the LED solar simulator and the solar radiation spectrum supports the relevance of this photocatalytic system under sunlight conditions (Table 1, Entry 5). Addition of BHT as a strong free radical quencher (Boulebd 2020; Durand et al. 2025) significantly reduced edible oil oxidation, supporting the involvement of carbon‐centered radicals generated through hydrogen abstraction (Table 1, Entry 6). Similarly, NaN_3_, a well‐established singlet oxygen scavenger (Li et al. 2001), markedly inhibited fatty acid oxidation, confirming the important contribution of ^1^O_2_ to the photooxidation process (Table 1, Entry 7).

**FIGURE 1:**
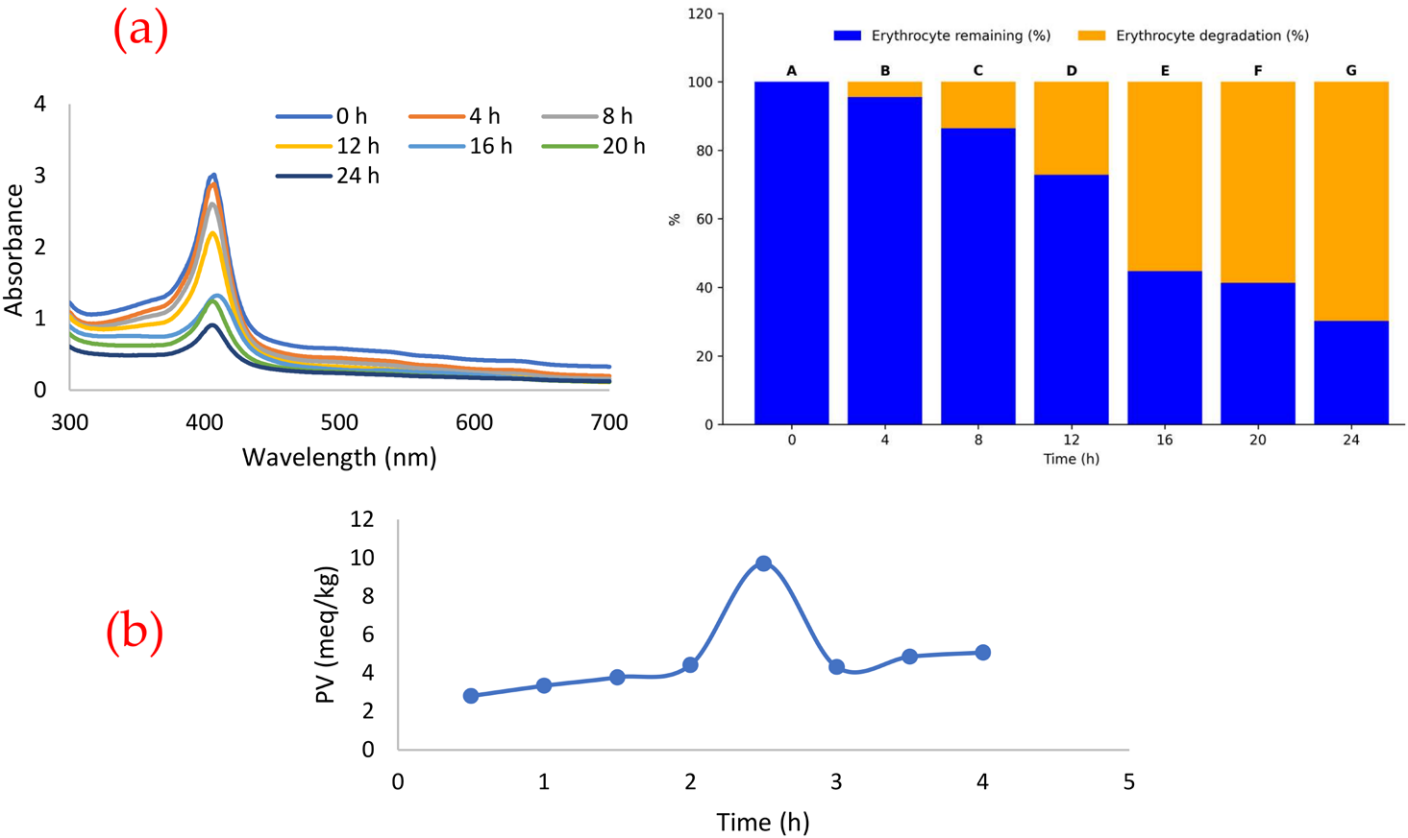
(a) UV–Vis spectral changes and erythrocyte remaining percentage of the edible oil–erythrocyte system during photooxidation under visible‐light irradiation. (b) Time‐dependent changes in (PV) during photooxidation. Different uppercase letters indicate statistically significant differences according to Duncan's multiple range test (*p* < 0.05).

**TABLE 1:** Photooxidation of edible oils using erythrocytes as a hemoglobin‐based photocatalytic system under various reaction conditions^a^.

Entry	Reaction condition	PV (meq O_2_/kg)
1	Edible oil + erythrocyte + air + visible light	9.7
2	Edible oil + air + visible light	Trace
3	Edible oil + erythrocyte + air	Trace
4	Edible oil + erythrocyte + visible light	Trace
5^b^	Edible oil + erythrocyte + air + sunlight	10.5
6^c^	Edible oil + erythrocyte + air + visible light + BHT	3.8
7^d^	Edible oil + erythrocyte + air + visible light + NaN_3_	3.1

^a^ Solution of edible oil (2 ⨯ 10^−2^ v/v) and erythrocyte (2 ⨯ 10^−2^ v/v) was illuminated by a solar simulator (276 power LED lamps [1 W, 2.3 V, 59,660 lux, 380–780 nm, 8.74 mW/cm^2^]) for 2.5 h at 25°C under 1 atm air bubble in a water/acetonitrile (9/1 v/v) solution.

^b^ The sunlight intensity was measured as 61,200 lux.

^c^ 0.0016 mmol NaN_3_ was used.

^d^ 0.0016 mmol BHT was used.

These scavenger experiments provide evidence for the coexistence of dual oxidative pathways involving both singlet oxygen and radical‐mediated reactions. Also, as shown in Figure 1b, the PVs increased progressively during the first 2.5 h of irradiation, reaching a maximum value of 9.7 meq kg^−1^. A subsequent slight decrease in PV was observed, which may be attributed to the decomposition of unstable primary hydroperoxides into secondary oxidation products. This trend indicates the continuous progression of lipid oxidative degradation within the oil matrix under the photocatalytic conditions.

### 3.2 Product‐Level NMR Evidence for Fatty Acid Oxidation Pathways

^1^H NMR analysis revealed light‐induced alterations in the fatty acid composition of edible oil in the presence of hemoglobin (Figure 2). To better understand the underlying oxidation mechanism, ^1^H NMR spectroscopy was employed to simultaneously detect multiple oxidation products and structural changes, including the formation of MDA (Guillén and Uriarte 2012; Hwang 2015, 2017; Liu et al. 2022; Ma et al. 2019). The vinylic proton signals (*δ* 5.2–5.4 ppm), which are susceptible to singlet oxygen‐mediated ene reactions (Bacellar and Baptista 2019), decreased by 31.2%, whereas the bis‐allylic proton signals (*δ* 2.7–2.8 ppm) decreased by 36.9%. The greater depletion of bis‐allylic protons is consistent with hydrogen‐abstraction reactions typically associated with radical‐mediated lipid oxidation pathways and may suggest the involvement of high‐valent heme intermediates (Davies and Guo 2014; Huang and Groves 2017). According to previous studies, these spectral changes indicate extensive oxidation at both double‐bond and bis‐allylic sites (Hwang 2017; Tynkkynen et al. 2012). Additionally, the appearance of a new proton resonance at *δ* 9.5 ppm (Figure 2b), confirms the formation of malondialdehyde (MDA), a well‐established marker of advanced lipid peroxidation and oxidative chain‐scission processes (Bacellar and Baptista 2019; Michel et al. 2008). Taken together, these spectral features demonstrated that ^1^O_2_‐mediated ene reactions (Huang and Groves 2017) and ferryl‐driven radical pathways (Huang and Groves 2017; Wilson and Reeder 2022) may operate concurrently in this photoreaction system.

**FIGURE 2:**
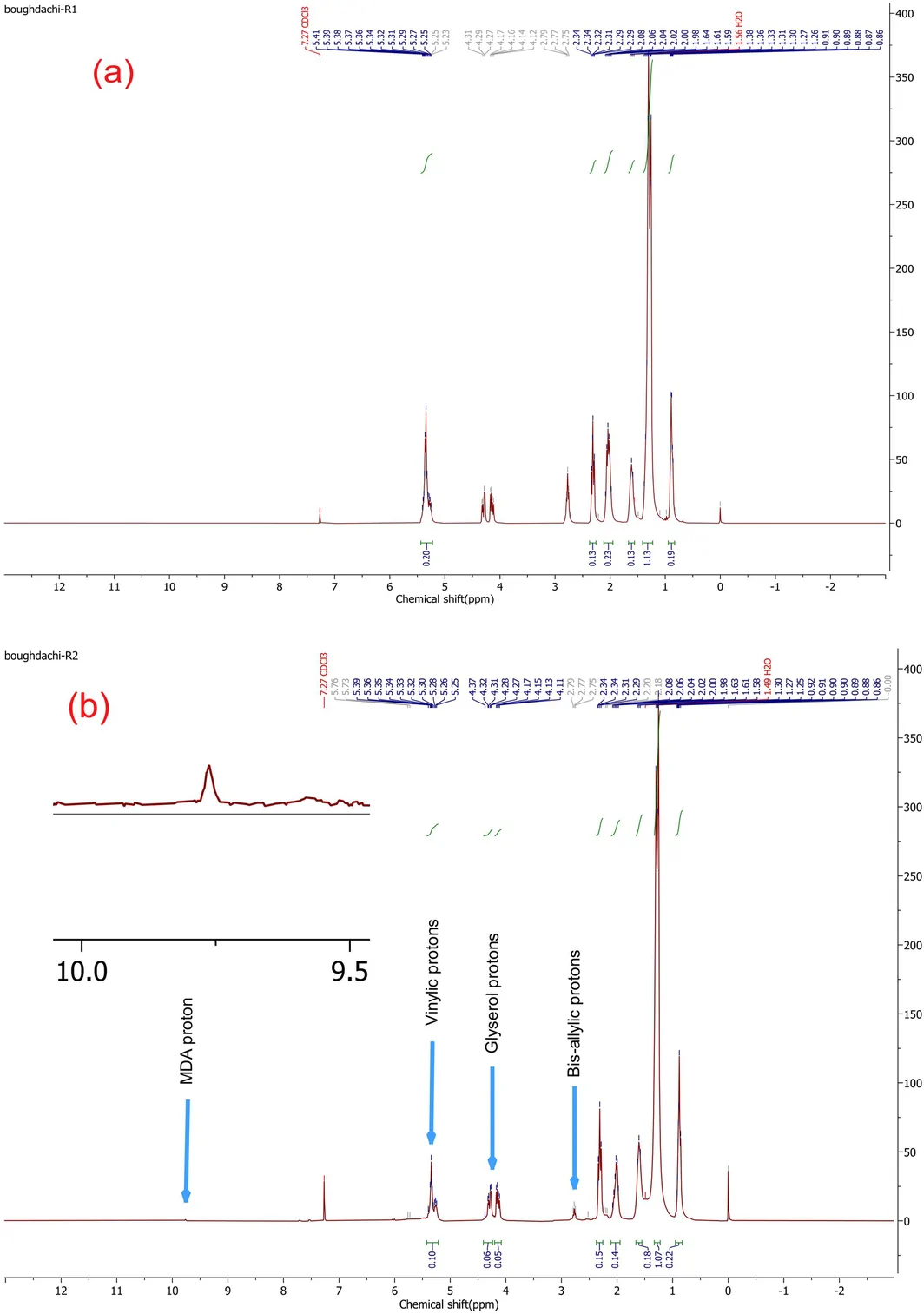
(a) ^1^H NMR spectra of edible oil before and (b) after photooxidation in the presence of erythrocytes.

### 3.3 Anthracene Bleaching as a Probe for Singlet Oxygen

In the Hb–edible oil system, singlet oxygen generation was directly evaluated using anthracene as a well‐known ^1^O_2_ probe (Joseph and Kumar 2023). The anthracene absorption band at 375 nm underwent approximately 33% photobleaching after 4 h of irradiation under visible light, indicating the formation of the corresponding endoperoxide product (Figure 3). Importantly, the addition of NaN_3_ as a recognized ^1^O_2_ quencher significantly inhibited the bleaching process. This suppression confirms that anthracene degradation was primarily driven by reactions involving ^1^O_2_ rather than alternative radical‐mediated pathways. These findings demonstrate that hemoglobin can act as an effective natural photosensitizing mediator for singlet oxygen generation under mild sunlight conditions.

**FIGURE 3:**
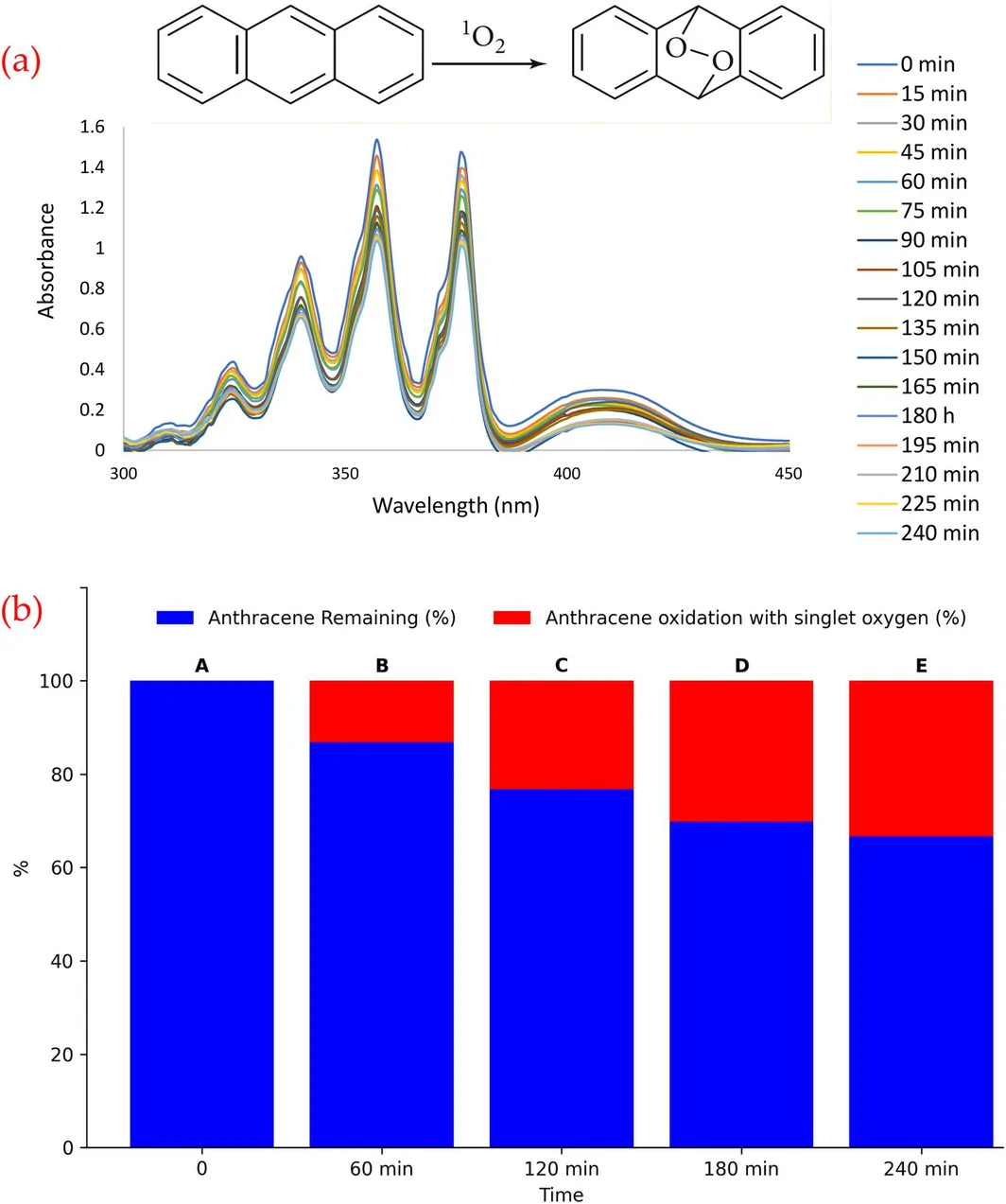
(a) UV–Vis absorption spectra of anthracene before and after irradiation in the presence of erythrocytes. (b) Schematic representation of anthracene photooxygenation with ^1^O_2_. Different uppercase letters indicate statistically significant differences according to Duncan's multiple range test (*p* < 0.05).

### 3.4 Solvent Effects on the Efficiency of Photooxidation

The addition of polar organic solvents significantly influenced the oxidation efficiency of edible oil in the presence of hemoglobin. The observed oxidation activity followed the order: acetonitrile > ethanol > acetone > methanol > DMSO (Table 2). This trend is consistent with the reported solvent‐dependent lifetimes of singlet oxygen, with ^1^O_2_ exhibiting a longer lifetime in acetonitrile (~65 μs) compared with DMSO (~19 μs) (Chen et al. 2001). Furthermore, highly coordinating solvents such as DMSO may inhibit the formation of reactive ferryl heme intermediates (HbFe^(IV)^═O) (Hajimohammadi et al. 2011) and suppress associated radical/ferryl‐mediated oxidation pathways (Sasak et al. 2024). Therefore, the lower oxidation efficiency observed in DMSO may be attributed to its stronger quenching effects toward both singlet oxygen and ferryl intermediates.

**TABLE 2:** Photooxidation of edible oil by hemoglobin in the presence of different solvents.^a^

Entry	Solvent	PV (meq O_2_/kg)
1	Acetonitrile	9.7
2	Ethanol	8.5
3	Acetone	7.6
4	Methanol	7.4
5	DMSO	2.6

^a^ Solution of edible oil (2⨯10^−2^ v/v) and erythrocyte (2 ⨯ 10^−2^ v/v) was illuminated by a solar simulator (276 power LED lamps [1 W, 2.3 V, 59,660 lux, 380–780 nm, 8.74 mW/cm^2^]) for 2.5 h at 25°C under 1 atm air bubble in a water/solvent (9/1 v/v) solution.

### 3.5 Comparison of Hemoglobin With Standard Oxidants

The photodegradation rate of edible oil based on PV (meq O_2_/kg) with different catalysts and oxidant followed the order Hb (9.7) > Rose Bengal (6.8) > KMnO_4_ (2.1). Rose Bengal acts as a selective generator of ^1^O_2_ (Fiegler‐Rudol et al. 2025), while KMnO_4_ mediates a classical electron‐transfer oxidation pathway (X. Wang et al. 2023). The superior performance of hemoglobin suggests that the synergistic contribution of ^1^O_2_ generation and ferryl‐mediated oxidation may results in enhanced oxidative activity compared with single‐pathway oxidants.

## 4 Discussion

### 4.1 Dual Oxidative Mechanism of Hemoglobin in Sunlight‐Induced Lipid Peroxidation

The present study was designed primarily to elucidate the mechanism of hemoglobin‐mediated sunlight‐induced lipid oxidation. The experimental results also provide preliminary evidence that this natural protein may be useful as a sustainable photosensitizer for lipid‐rich wastewater treatment. The findings of the present study suggest that hemoglobin promotes lipid oxidation through two possible pathways: ^1^O_2_‐mediated oxidation and radical‐mediated reactions. The contribution of singlet oxygen is supported by several observations, including anthracene bleaching, inhibition by NaN_3_ (a known ^1^O_2_ quencher), solvent‐dependent oxidation patterns consistent with ^1^O_2_ lifetime differences, and the formation of MDA accompanied by selective oxidation of vinylic sites. These results are reliable with established ^1^O_2_–driven lipid oxidation mechanisms in porphyrin‐based systems. In contrast, the formation of a ferryl (HbFe^(IV)^═O)‐type species was not directly confirmed in the present study. Its possible involvement is proposed based on indirect evidence, including the inhibitory effect of BHT, the decrease in bis‐allylic proton signals observed by ^1^H NMR, and the influence of DMSO on oxidation behavior. These observations are consistent with hydrogen‐abstraction reactions typically associated with high‐valent heme species. This interpretation is supported by previous studies on related heme systems, where ferryl intermediates have been detected, including UV–Vis observation of (TMPFe^(IV)^═O)^2+^ in metalloporphyrin models (Hajimohammadi et al. 2011) and mass spectrometric detection of Fe^(IV)^═O–protoporphyrin IX^+^ under oxidative conditions (Hajimohammadi, Verjani, et al. 2018). Also, hemoglobin exhibited higher oxidative efficiency than Rose Bengal and KMnO_4_, suggesting that its activity arises from the combined action of singlet oxygen chemistry and additional radical pathways rather than a single dominant mechanism.

### 4.2 Proposed Pathway for Hemoglobin‐Mediated Photodegradation of Edible Oil

The proposed dual oxidative mechanism underlying hemoglobin‐mediated photodegradation of edible oil is illustrated in Figure 4. Upon light exposure, hemoglobin promotes lipid oxidation through the combined contribution of ^1^O_2_‐mediated and radical‐type pathways. The ^1^O_2_ pathway is proposed to initiate oxidation through ene‐type reactions at vinylic positions (*δ* 5.2–5.4 ppm, ^1^H NMR), leading to hydroperoxide formation and weakening of the lipid structure. In parallel, a ferryl heme–associated pathway may contribute to oxidation at bis‐allylic positions (*δ* 2.7–2.8 ppm, ^1^H NMR) through hydrogen abstraction reactions, promoting secondary radical reactions and fatty acid chain degradation. ^1^H NMR analysis also revealed the formation of MDA as an aldehydic product, demonstrating the cleavage of unsaturated fatty acids into smaller, more polar oxidation products. These compounds are products of lipid degradation and serve as molecular indicators of the progressive breakdown of the oil matrix during photooxidation. Overall, the synergistic contribution of ^1^O_2_‐mediated oxidation and possible ferryl‐associated radical processes may explain the high photodegradation efficiency of hemoglobin and supports its potential application in natural or biomimetic photo‐oxidative systems. In addition to the changes observed in the unsaturated regions, the glycerol proton signals at ~4.10 ppm exhibited noticeable perturbation, suggesting degradation of the –CH_2_–O–CO– ester linkages within the triglyceride backbone. Such alterations are characteristic of advanced lipid peroxidation, where radical‐driven β‐scission cleaves the ester bond connecting fatty acids to glycerol. This evidence is consistent with established mechanisms of lipid oxidation, in which high‐valent heme species such as ferryl intermediates can promote hydrogen abstraction and radical‐mediated oxidation processes that may contribute to triglyceride fragmentation (Kamal‐Eldin 2003; Porter et al. 1995). In contrast, singlet oxygen primarily reacts with C═C bonds through selective oxidation pathways and has limited reactivity toward ester moieties.

**FIGURE 4:**
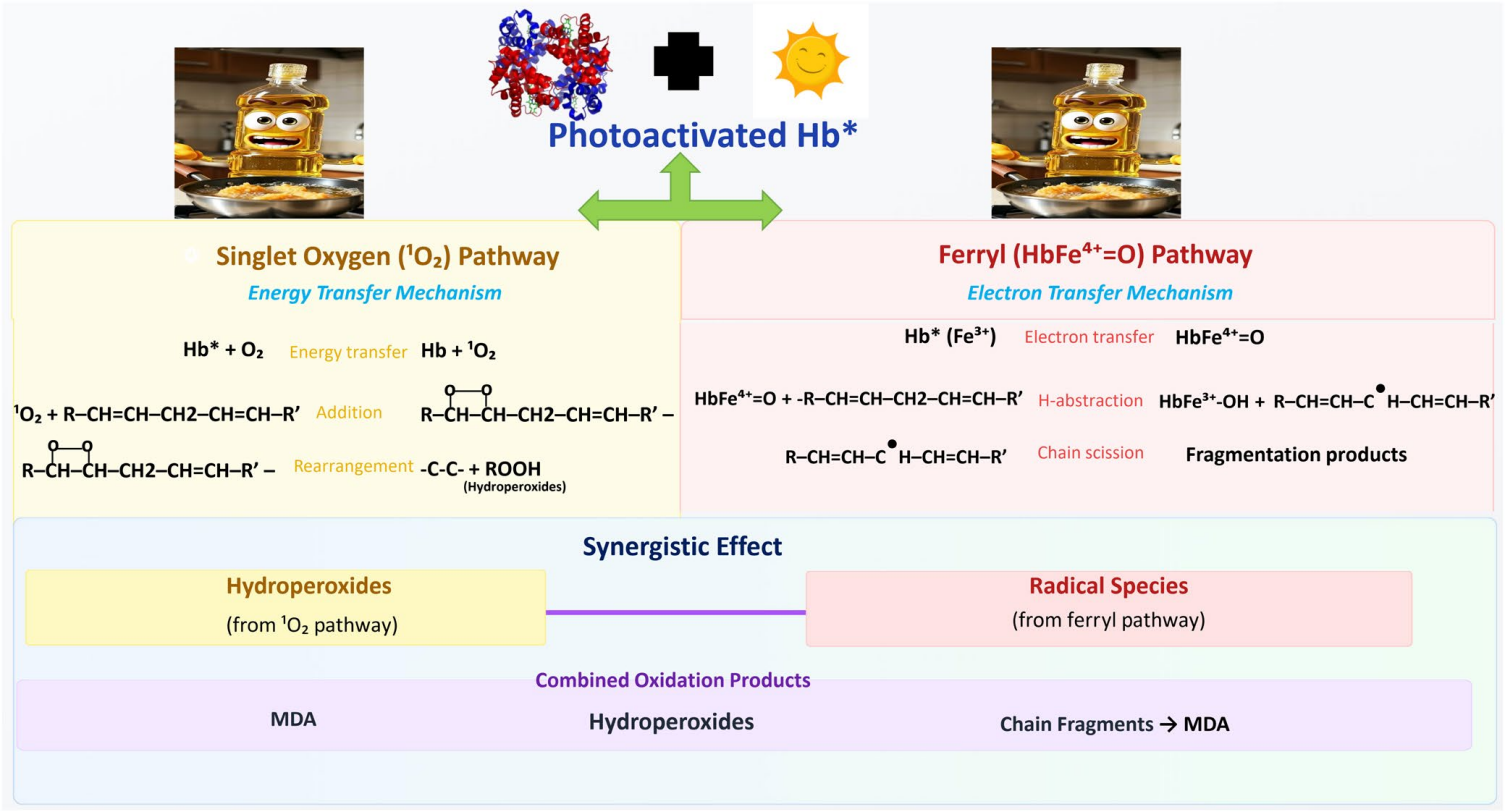
Proposed mechanisms for hemoglobin‐mediated photooxidation of edible oil involving singlet oxygen generation and ferryl/radical oxidation pathway.

### 4.3 Environmental Implications

Hemoglobin is a biodegradable, food‐derived protein obtained as a by‐product of the meat processing industry. Its abundance, low cost, and renewable nature make it a promising material for valorizing animal‐processing waste within circular food systems. As a light‐responsive mediator, it enables sunlight‐driven oxidation of lipid‐rich effluents without the need for synthetic oxidants, offering a greener and more resource‐efficient approach for food industry wastewater treatment. These features highlight its potential for sustainable lipid degradation and waste management applications.

### 4.4 Study Limitations and Future Perspectives

Several limitations of the present study should be considered. First, this study used a simplified edible oil model rather than real industrial effluents; therefore, the influence of complex wastewater compositions on oxidation efficiency requires further investigation. Second, the long‐term stability of hemoglobin under continuous irradiation and the feasibility of scale‐up, including light penetration, oxygen transfer, reactor design, and protein recovery, remain to be evaluated. In addition, although the findings support a possible ferryl‐associated pathway, direct spectroscopic confirmation was not performed and should be addressed in future studies. Despite these limitations, this work highlights the potential of hemoglobin as a food‐derived photosensitizer for sustainable sunlight‐driven lipid oxidation and wastewater treatment applications.

## 5 Conclusions

This study primarily provides mechanistic insight into sunlight‐induced lipid oxidation mediated by erythrocyte‐derived hemoglobin. The collective experimental evidence supports the coexistence of singlet oxygen‐mediated oxidation and a likely radical‐associated pathway, although direct spectroscopic evidence for ferryl intermediates was not obtained. Beyond advancing understanding of hemoglobin‐mediated photooxidation, the results also suggest that blood‐derived proteins could serve as low‐cost, sustainable photosensitizers for future treatment of lipid‐rich wastewater. The synergistic interaction of these pathways promotes extensive oxidation of unsaturated fatty acids, resulting in hydroperoxide formation, MDA generation, and progressive lipid degradation. Beyond providing mechanistic insight into heme‐mediated lipid oxidation relevant to food storage and processing, these findings highlight the potential of food‐derived proteins as sustainable functional materials. Given its biodegradability, abundance as a meat industry by‐product, and low cost, hemoglobin represents a promising candidate for valorization within circular food systems. Future work should focus on protein stabilization, immobilization strategies, and bioinspired hybrid materials to improve photooxidative performance and enable scalable, solar‐driven applications for the treatment of lipid‐rich effluents in food processing industries.

## Author Contributions

**Mahdi Hajimohammadi:** conceptualization, investigation, funding acquisition, writing – original draft, writing – review and editing, visualization, validation, methodology, software, formal analysis, project administration, data curation, supervision, resources. **Fadhel H. Faraj:** resources, writing – review and editing, funding acquisition, project administration. **Mona Boghdachi:** software, formal analysis, methodology, data curation. **Asawer A. Alwasiti:** conceptualization, resources, project administration, writing – review and editing. **Zainab Y. Shnain:** writing – review and editing, resources, project administration, funding acquisition.

## Funding

This work was supported by Kharazmi University, 404703.

## Ethics Statement

Defibrinated sheep blood used in this study was commercially obtained from Kimiakavoshazma Company (Iran). No animals were directly handled, housed, sampled, or subjected to experimental procedures by the authors. The study involved only commercially supplied biological material and did not include live animal experimentation. Therefore, institutional animal ethics approval was not required according to the applicable institutional guidelines.

## Conflicts of Interest

The authors declare no conflicts of interest.

## Data Availability

The data that support the findings of this study are available from the corresponding author upon reasonable request.
